# Preventing Cushing: Iatrogenic Cushing Syndrome due to Ritonavir-Fluticasone Interaction

**DOI:** 10.7759/cureus.1484

**Published:** 2017-07-17

**Authors:** Fasil Tiruneh, Ahmad Awan, Abiot Didana, Saumil Doshi

**Affiliations:** 1 Department of Internal Medicine, Howard University Hospital; 2 Cardiovascular Technician, Inova Mount Vernon Hospital

**Keywords:** highly active antiretroviral therapy (haart), human immunodeficiency virus (hiv), cytochrome p450, cushing's syndrome

## Abstract

Ritonavir is commonly used in low doses to boost plasma levels of protease inhibitors in patients with human immunodeficiency virus (HIV) infections. It is also a potent inhibitor of cytochrome P450. We present a 50-year-old African American male with past medical history of HIV on highly active antiretroviral therapy (HAART), which also included ritonavir and long standing asthma that has been treated with inhaled fluticasone, who presented with back pain. He had central obesity, prominent abdominal striae and wasted extremities on physical examination. Laboratory tests showed low morning serum cortisol and suboptimal cosyntropin test consistent with adrenal insufficiency. Computed tomography (CT) of the spine showed a fracture of inferior endplate of the lumbar (L3) vertebra. The cause of osteoporosis is believed to be iatrogenic Cushing syndrome caused by enhanced levels of inhaled fluticasone effects secondary to inhibition of cytochrome P450. The patient was managed surgically and fluticasone was discontinued.

## Introduction

Ritonavir, a potent inhibitor of the hepatic cytochrome P450 is commonly used in low doses to boost plasma levels of other protease inhibitors in patients with human immunodeficiency virus (HIV) [1]. Intranasal and inhaled corticosteroids are widely used for the treatment of allergic rhinitis and asthma. Inhaled steroids do not usually lead to systemic adverse events since their plasma concentrations are low due to extensive first-pass metabolism and clearance by cytochrome P450 3A4 (CYP3A4). However, the coadministration of ritonavir with inhaled (or intranasal) corticosteroids may result in an increase in the plasma corticosteroid levels due to the potent CYP3A4 inhibition by ritonavir. This may cause iatrogenic Cushing's syndrome with adrenal suppression [2]. Osteoporosis is one of the commonest and severe adverse effects of glucocorticoid excess and one of the major limitations to long-term glucocorticoid therapy [3]. Informed consent was obtained for this study.

## Case presentation

**Figure 1:**
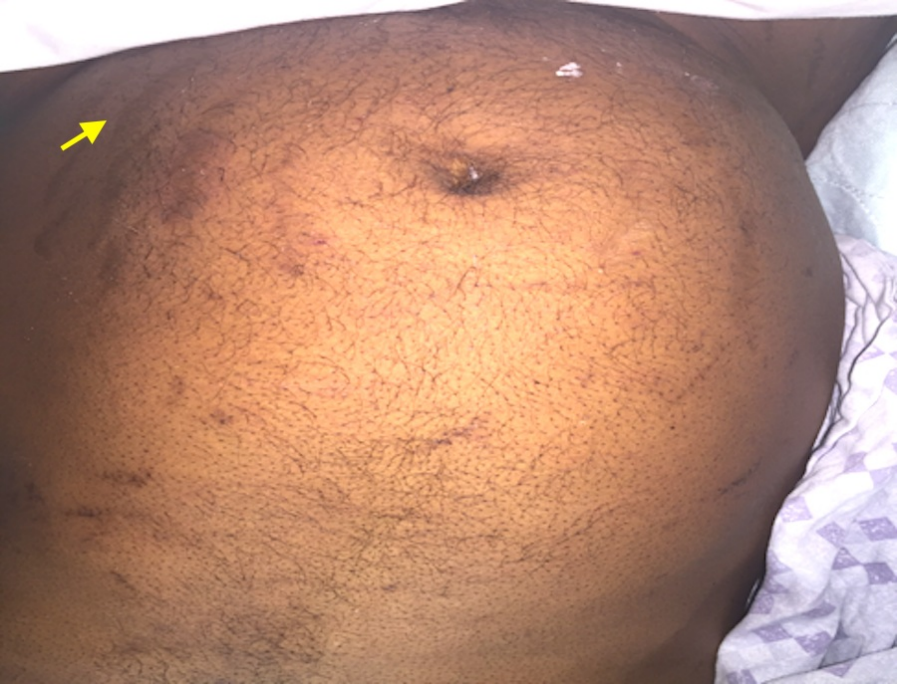
Image showing abdominal striae ( yellow arrow)

**Figure 2:**
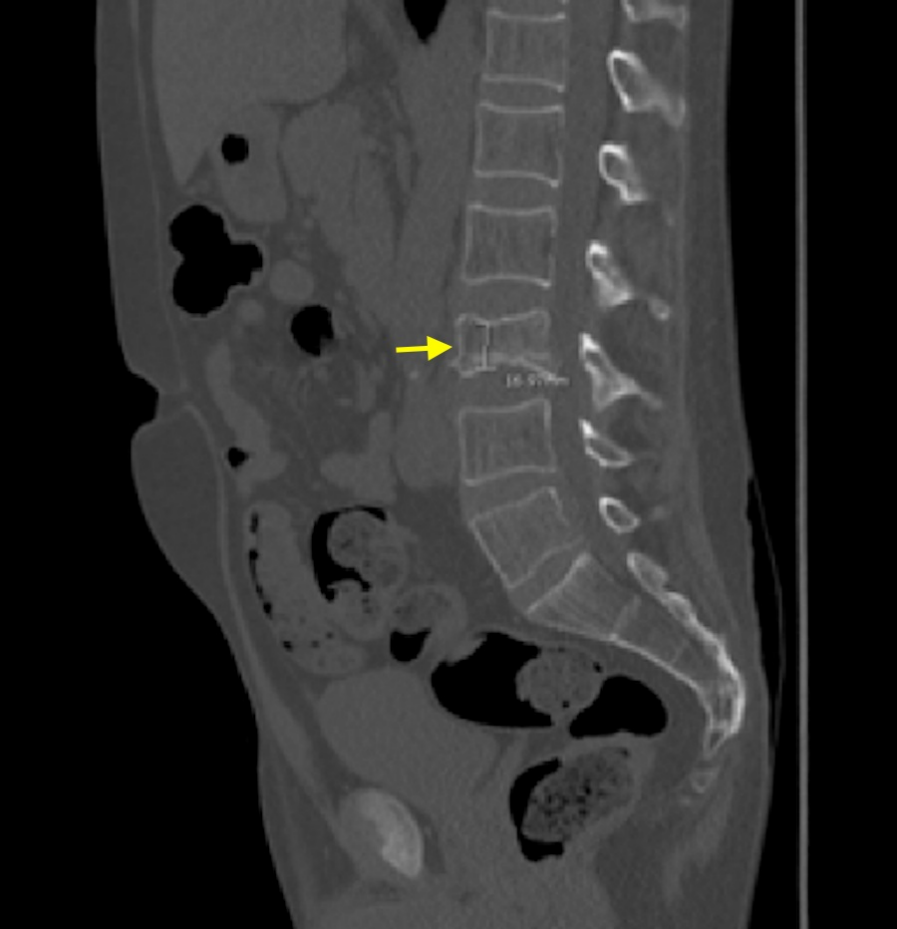
Image showing the computed tomography of generalized osteoporosis and compression fracture of lumbar (L3) vertebral body (yellow arrow)

A 50-year-old African American male patient with a past medical history of human immunodeficiency virus (HIV) on highly active antiretroviral therapy (HAART) for 15 years and long standing asthma that has been treated with inhaled fluticasone 200mcg/day and as needed albuterol for more than 15 years presented with sharp lower back pain of three days' duration that started while he was lifting heavy weight. For his HIV, he had been initially treated with abacavir and efavirenz which was later changed to Truvada one tablet, atazanavir 300mg, and ritonavir 100mg oral daily because of virologic failure. The patient also had recent onset type two diabetes mellitus and hypertension. On examination, he had central obesity with prominent abdominal striae (Figure 1) and wasted extremities. Localized tenderness was noted at the lower lumbar vertebral body. A computed tomography (CT) of the spine showed compression fracture of inferior endplate of L3 vertebra, diffuse osteoporosis, and aseptic necrosis of bilateral femoral heads (Figure 2).

Laboratory tests showed low morning serum cortisol level (0.2 ug/L [normal 5-25 nmol/L) and suboptimal cosyntropin stimulation test consistent with adrenal suppression (8.14, 12.65, and 13.25 nmol/L at zero, 30, and 60 minutes respectively ). Luteinizing hormone (LH) and follicular stimulating hormone (FSH) were 3.7 and 7.9 IU/mL respectively (normal FSH 1.5 to 12.4 IU/L and LH 1.8 to 8.6 IU/L). Celiac screening with tissue transglutaminase and anti-gliadin antibodies were negative. His thyroid function test was normal. The patient was started on hydrocortisone to prevent adrenal crises. Decompressive lumbar laminectomy with bilateral facetectomies and foraminotomies and arthrodesis at L2-L3 and L3-L4 were done. Fluticasone was discontinued and he was discharged with the bronchodilator.

## Discussion

Cushing disease is caused by adrenal, pituitary adenomas, ectopic tumors secreting adrenocorticotropic hormone (ACTH), or iatrogenic causes. It is associated with an increased risk of cardiovascular, metabolic, respiratory, psychiatric complications, osteoporosis, and infections leading to high rates of morbidity and mortality. The prevalence of Cushing’s disease is of 40:1,000,000 [4]. More than 10 million Americans receive pharmacologic doses of glucocorticoids each year making iatrogenic Cushing's syndrome resulting from long-term use of exogenous glucocorticoids the most common cause of Cushing's syndrome [3].

Iatrogenic Cushing's is one of the leading cause of the syndrome and should be taken into consideration and excluded in any patient who presented with sign and symptom of Cushing’s disease. Screening tests are used in Cushing’s disease to identify the excessive secretion of cortisol, these include ACTH and cortisol levels [4]. However, detailed history including medication reconciliation and physical examination with a high index of suspicion is needed to diagnose iatrogenic Cushing's Tests to demonstrate the suppression of pituitary-adrenal axis and appropriate targeted tests to rule out complications are necessary. 

Drugs that have been reported to result in hypercortisolism are glucocorticoids, megestrol acetate and herbal preparations that contain glucocorticoids [5]. The coadministration of ritonavir and fluticasone at the recommended doses is known to cause iatrogenic Cushing's syndrome with adrenal suppression. The clinical manifestation of Cushing's can be confused with HIV associated lipodystrophy [2]. 

It is described that the risk of osteoporosis and osteonecrosis associated with exogenous glucocorticoid use can occur in the absence of low mineral bone density [3]. Our patient was noted to have the complications including hypertension, diabetes mellites, osteoporosis, and osteonecrosis. Despite several clinic visits, his medication interaction was overlooked and finally, he presented with osteoporotic fracture. We suggest that high clinical suspicion is needed for early diagnosis and prevention of Addisonian crises.

Due to this side effect profile of ritonavir-fluticasone, when ritonavir is needed as a booster, a low-dose budesonide or beclomethasone can be used cautiously along with close monitoring for symptoms of adrenal insufficiency upon discontinuation of inhaled corticosteroids [6]. The option of replacing ritonavir based therapy can also be considered in patients taking fluticasone. In the SPIRAL study, raltegravir demonstrated non-inferior efficacy and improved lipid profile when ritonavir boosted protease inhibitor therapy was replaced by raltegravir [7].

## Conclusions

Fluticasone treatment should be avoided in patients who are treated with ritonavir. Other inhaled glucocorticoids, such as beclomethasone and budesonide appear to be safer options because of their lower binding affinity for glucocorticoid receptors and shorter elimination of half-life. However, they are also cytochrome P450 3A4 (CYP3A4) substrates and similar cases have been described. Caution should be used when any inhaled glucocorticoid is combined with ritonavir. Alternative therapeutic options for asthma control such as oral montelukast or bronchodilators alone should be considered.
